# Identification of flucloxacillin-modified hepatocellular proteins: implications in flucloxacillin-induced liver injury

**DOI:** 10.1093/toxsci/kfad015

**Published:** 2023-02-14

**Authors:** Serat-E Ali, James C Waddington, Adam Lister, Rowena Sison-Young, Robert P Jones, Adeeb H Rehman, Chris E P Goldring, Dean J Naisbitt, Xiaoli Meng

**Affiliations:** Department of Molecular & Clinical Pharmacology, University of Liverpool, Sherrington Buildings, Ashton Street, Liverpool, L69 3GE, UK; Department of Molecular & Clinical Pharmacology, University of Liverpool, Sherrington Buildings, Ashton Street, Liverpool, L69 3GE, UK; Department of Molecular & Clinical Pharmacology, University of Liverpool, Sherrington Buildings, Ashton Street, Liverpool, L69 3GE, UK; Department of Molecular & Clinical Pharmacology, University of Liverpool, Sherrington Buildings, Ashton Street, Liverpool, L69 3GE, UK; Department of Hepatobiliary Surgery, Aintree University Hospital, Liverpool University Hospitals, NHS Foundation Trust, Liverpool, UK; Department of Hepatobiliary Surgery, Aintree University Hospital, Liverpool University Hospitals, NHS Foundation Trust, Liverpool, UK; Department of Molecular & Clinical Pharmacology, University of Liverpool, Sherrington Buildings, Ashton Street, Liverpool, L69 3GE, UK; Department of Molecular & Clinical Pharmacology, University of Liverpool, Sherrington Buildings, Ashton Street, Liverpool, L69 3GE, UK; Department of Molecular & Clinical Pharmacology, University of Liverpool, Sherrington Buildings, Ashton Street, Liverpool, L69 3GE, UK

**Keywords:** covalent binding, flucloxacillin DILI, membrane transporters, hepatocytes

## Abstract

Flucloxacillin is a β-lactam antibiotic associated with a high incidence of drug-induced liver injury. Although expression of HLA-B*57:01 is associated with increased susceptibility, little is known of the pathological mechanisms involved in the induction of the clinical phenotype. Irreversible protein modification is suspected to drive the reaction through the provision of flucloxacillin-modified peptides that are presented to T-cells by the protein encoded by the risk allele. In this study, we have shown that flucloxacillin binds to multiple proteins within human primary hepatocytes, including major hepatocellular proteins (hemoglobin and albumin) and mitochondrial proteins. Inhibition of membrane transporters multidrug resistance-associated protein 2 (MRP2) and P-glycoprotein (P-gp) appeared to reduce the levels of covalent binding. A diverse range of proteins with different functions was found to be targeted by flucloxacillin, including adaptor proteins (14-3-3), proteins with catalytic activities (liver carboxylesterase 1, tRNA-splicing endonuclease subunit Sen2, All-trans-retinol dehydrogenase ADH1B, Glutamate dehydrogenase 1 mitochondrial, Carbamoyl-phosphate synthase [ammonia] mitochondrial), and transporters (hemoglobin, albumin, and UTP-glucose-1-phosphate uridylyltransferase). These flucloxacillin-modified intracellular proteins could provide a potential source of neoantigens for HLA-B*57:01 presentation by hepatocytes. More importantly, covalent binding to critical cellular proteins could be the molecular initiating events that lead to flucloxacillin-induced cholestasis Data are available via ProteomeXchange with identifier PXD038581.

## Introduction

Flucloxacillin, a β-lactam antibiotic primarily utilized in the treatment of staphylococcal infection, is frequently implicated in drug-induced cholestatic liver injury. The isolation and characterization of flucloxacillin reactive IgE as well as CD4+ and CD8+ T-cells from patients with drug-induced liver injury (DILI) suggest the immune system is the protagonist in flucloxacillin-DILI (Baldo *et al.*, 1995; Monshi *et al.*, 2013). Coupled with a strong association with the HLA-B*57:01 allele, the adaptive immune system has been proposed as the prevailing mechanism for flucloxacillin-mediated DILI (Daly *et al.*, 2009). The modification of endogenous proteins as well as the identification of neoantigens for human leukocyte antigen (HLA) presentation in a flucloxacillin-treated lymphoblastoid cell line (C1R) over-expressing HLA-B*57:01 augments the immune-mediated theory, proposing a significant role of neo-antigen formation in disease promotion (Waddington *et al.*, 2020b). Despite the characterization of such drug-protein adducts, in vitro immune responses and strong HLA association, little is known about the molecular mechanisms which lead to the initiation of this pathway. We have recently shown that flucloxacillin has a strong propensity to bind to proteins localized within the bile canaliculi (BC) regions of differentiated HepaRG cells, the primary site of tissue injury in patients with flucloxacillin induced-cholestasis (Waddington *et al.*, 2020a). Covalent binding of flucloxacillin at the BC is primarily mediated by membrane transporters such as multidrug resistance-associated protein 2 (MRP2) and P-glycoprotein (P-gp). Binding of flucloxacillin at these BC regions and within hepatocytes may provide a cradle for neo-antigen formation for subsequent HLA presentation and T-cell activation. We hypothesized that disruption of membrane transporters crucial for flucloxacillin efflux results in the formation of novel antigenic determinants.

Previously, Burban *et al.* have shown that flucloxacillin induced direct cholestatic effects in hepatocytes through activation of HSP27 that led to downstream activation of the PI3K/AKT signaling pathway (Burban *et al.*, 2017). This nonimmune-mediated cholestasis may induce danger signals such as Damage-associated molecular patterns (DAMPs) released by stressed cells (Ogese *et al.*, 2017) regulating the initial flucloxacillin-specific immune activation, thereby determining the progression and severity of liver injury. However, the events leading up to activation of these signaling pathways are largely unknown and yet to be defined. it is conceivable that transport-dependent cellular accumulation of flucloxacillin may either induce cellular stress or covalent binding to certain hepatocellular proteins, leading to activation of HSP27 associated signaling pathways and subsequently flucloxacillin-induced cholestasis. This study was therefore performed to investigate the effect of cell membrane transporters on the transportation of flucloxacillin and covalent binding of flucloxacillin to proteins using the immortalized hepatic cell line HepaRG and primary human hepatocytes (PHH).

## Materials and methods

### Chemicals

Flucloxacillin (Wockharat) was gifted from collaborators. Unless otherwise described, chemicals were purchased from Merck (Gillingham, Dorset, United Kingdom).

### Anti-flucloxacillin antibody production

Antibody production was performed by Kaneka Eurogentec S.A. (Belgium) using a speedy 28-polyclonal package using Ovalbumin-flucloxacillin conjugates. Ovalbumin (OVA) (Imject™, ThermoScientific, UK) was used as a protein carrier to generate drug-protein conjugates for antibody production. OVA was made up to 1 mM solution in phosphate buffer (13.08 mM KH2PO4, 62.27 mM K2HPO4, pH 7.4) and was incubated with flucloxacillin at a 1:100 molar ratio (protein to flucloxacillin) at 37°C for at least 16 h. The drug-protein conjugates were purified to remove free flucloxacillin using spin filters with a 3 kDa molecular weight cut off (Amicon Ultra, Merck, Dorset, UK) following manufacturer’s instructions. Binding of flucloxacillin to OVA was characterized by mass spectrometry analysis (Waddington *et al.*, 2020b). The speedy 28-polyclonal package was used for the production of a high antibody titer with high antibody affinity. Injections (100 µg/injection) are administered to two rabbits at days 0, 7, 10, and 18. A pre-immune bleed is taken on day 0, a medium bleed on day 21 and a final bleed on day 28. The bleeds are supplied as serum containing polyclonal antibody. Subsequent ELISA is performed by Eurogentec and in house (in addition to Western blot analysis). Detailed information is available online (eurogentec.com; last accessed February 15, 2023). The specificity of anti-flucloxacillin antibodies was extensively validated using both ELISA and Western blot analysis using a diverse range of antigens including protein-conjugates formed by other β-lactams, unmodified single proteins, and cellular proteins (Waddington *et al.*, 2020a; Waddington *et al.*, 2020b).

### Cell culture and viability assessment

PHHs were isolated from liver biopsies taken from consenting donors. Liver biopsies were collected from liver resections of varying etiologies conducted at the University of Liverpool teaching hospital, Aintree, Liverpool, Merseyside. Written informed consent was obtained from donors to partake in the research study according to the Declaration of Helsinki and it has been approved by the local Liverpool research ethics committee. PHHs were isolated using a perfusion-digestion method (Heslop *et al.*, 2017). Hepatocytes were then cultured in Williams E media supplemented with L-glutamine (2 mM), penicillin (100 μg/mL), streptomycin (100 U/mL), insulin–transferrin–selenium (100×), and dexamethasone (1 μM/mL) on either precoated collagen plates or collagen-coated glass cover slides. To assess toxicity, PHHs were plated in collagen-coated, flat-bottomed 96-well plates (ThermoScientific, UK) and incubated with various concentrations of flucloxacillin for 24 h at 37°C, 5% CO_2_. The viability of the cells was then analyzed using CellTiter-Glo^®^ Cell Viability Assay (Promega, UK), as a luminescence value in comparison to an untreated control.

### Detection of intracellular flucloxacillin-modified proteins by western blotting

PHHs were harvested and washed with HBSS (Sigma Poole, UK) prior to being pelleted and snap frozen. Cell pellets were lysed (7.0M urea, 2.0M thiourea, 4% CHAPS, 40 mM Tris base, and 1% DTT) and soluble lysates were collected for protein quantification. Proteins were denatured and reduced by heating to 100°C for 10 min in Laemmli sample buffer (Sigma, Poole, UK). One hundred micrograms of protein from human primary hepatocytes cell lysates were separated in two dimensions on a 10% SDS-polyacrylamide gel for both Coomassie-stained gels and Western blots. The first dimension was performed by rehydrating IPG strips (pH 4-7) with sample in rehydration solution and separating based on pH using the Multiphor Electrophoresis System (GE Healthcare, MA, USA; Kitteringham *et al.*, 2003). For Western blotting, gels were transferred onto nitrocellulose membrane by electroblotting. The nitrocellulose membrane was washed in deionized water and blocked in tris/saline/tween (TST) buffer (150 mM NaCl, 10 mM Tris-HCl, 0.05% Tween 20, pH 8.0) containing 2% nonfat dry milk (Bio-Rad) for 1 h at room temperature. The nitrocellulose was washed in TST and incubated in primary polyclonal rabbit-anti-flucloxacillin antibody (Waddington *et al.*, 2020b; custom order, Eurogentec, Belgium) in TST containing 2% nonfat dry milk for 1 h. The nitrocellulose membrane was washed in TST repeatedly and incubated with horseradish peroxidase-conjugated goat-anti-rabbit secondary antibody (Dako, Agilent, CA, USA) for 1 h. After further washing with TST, signal was detected using enhanced chemiluminescence (Western Lightning, PerkinElmer Life and Analytical Sciences, Waltham, MA).

### Detection of flucloxacillin modified proteins by immuno-cytochemistry

Cells were cultured in the presence of flucloxacillin (1.5 mM) and adhered to glass coverslips using Cell-Tak (Corning, MA, USA). Cells were washed with PBS (pH 8.0) and fixed using 4% paraformaldehyde. Cells were permeabilized (0.004% Tween 20, 0.025% Triton-X-100, PBS) for 30 min and blocked with bovine serum albumin (5% in permeabilization buffer) for 1 h at room temperature. Subsequently, blocking buffer containing polyclonal rabbit-anti-flucloxacillin antibody at 4°C was added overnight. After washing with permeabilization buffer, goat anti-Rabbit IgG secondary antibody (Alexa Fluor 488, ThermoScientific, MA, USA) was applied for 1 h. After further washes, the cells were incubated in Hoechst 33342 (ThermoScientific, MA, USA) and Alexa-Fluor-568-phalloidin (ThermoScientific, MA, USA) for nuclear and f-actin staining, respectively. Cover slips were mounted onto glass slides with Pro-Long Gold (ThermoScientific, MA, USA) and sealed. Images were taken using a Carl Zeiss Axio Observer microscope with Apoptome using 40× oil objective.

### Immunoaffinity enrichment of flucloxacillin-modified peptides

Hepatocytes treated with 1.5 mM flucloxacillin for 24 h were pelleted and lysed using lysis buffer (7.0M urea, 2.0M thiourea, 4% CHAPS, 40 mM Tris base, and 1% DTT). Cell lysates were cleared using centrifugation, reduced (10 mM DTT, room temperature for 20 min), and alkylated (55 mM IAA, room temperature in the dark, for 20 min). Digestion was performed using sequencing grade modified trypsin (Promega, UK) overnight at 37°C. Flucloxacillin-modified peptides were first enriched prior to LC-MS/MS analysis of solid-phase-bound polyclonal anti-flucloxacillin antibody according previous protocols (Waddington *et al.*, 2020b). Briefly, tryptic peptide mixture was incubated with protein A beads (Repligen, MA, USA) conjugated with polyclonal anti-flucloxacillin antibody for 1 h at 4°C, followed by acid elution with 10% acetic acid. The eluent was further purified using C18 ZipTips (Millipore, UK) and dried in a centrifugal concentrator (Eppendorf Speedvac, UK) prior to LC-MS/MS analysis.

### Identification of flucloxacillin modified proteins via mass-spectrometry

Samples were reconstituted in 2% ACN, 0.1% FA (v/v) prior to analysis using a Triple TOF 6600 mass spectrometer (Sciex, UK) delivered into the instrument using an Eksigent NanoLC Ultra HPLC system. Samples were injected onto a nanoACQUITY UPLC Symmetry C18 Trap Column (P/N Waters, MA, USA) and washed for 10 min at 2 µL/min with 0.1% FA. A gradient from 1.6% ACN/0.1% FA to 95% ACN/0.1% FA was applied over 95 min at a flow rate of 300 nL/min through a Peptide BEH C18 nanoACQUITY Column (Waters, MA, USA). MS was operated as described in previous methods (Meng *et al.*, 2016).

### Analysis of tryptic peptides

LC-MS/MS data were searched using PEAKS X Pro (Version 10.6 build 20201015) with the following specifications: incorporating enzymatic cleavage restriction for Trypsin (maximum 3 missed cleavages, allowing for nonspecific cleavage), fixed modification carbamidomethylation of cysteine, Variable modifications—methionine oxidation (+15.99), asparagine and glutamine deamidation (+0.98), flucloxacillin modification of lysine and arginine (+453.06, incorporating a neutral loss of 159.04 on fragmentation). The maximum number of variable posttranslational modifications per peptide was 3. False discovery rate was estimated with decoy fusion. Sequences annotated with a flucloxacillin modification were considered valid if they contained the diagnostic 160.04 ion. Flucloxacillin-modified peptides were also manually identified using characteristic fragment ions of m/z at 160.04, 295.03, and 454.06; diagnostic ions indicative of the presence of a covalently linked flucloxacillin molecule. Manual annotation of MS/MS spectra was performed to identify the peptides and subsequent NCBI Blast searches were used to identify the native protein sources. The mass spectrometry proteomics data have been deposited to the ProteomeXchange Consortium (http://proteomecentral.proteomexchange.org; last accessed February 15, 2023) via the PRIDE partner repository (Perez-Riverol *et al.*, 2022) with the dataset identifier PXD PXD038581 and 10.6019/PXD038581.

### Prediction of binding affinity of hemoglobin peptides to HLA-B*57:01

Hemoglobin protein sequence (FASTA format) was exported from UniProtKB/SwissProt database. Predicted binding affinities were calculated by NetMHC4.0 (Andreatta and Nielsen, 2016; Nielsen *et al.*, 2003). Peptide length was specified as 9-mers and HLA-B*57:01 was specified as the selected allele. Quantitative affinity measurements that have IC50 value of < 500 nM for HLA-B*57:01 were considered as good binders (Zhao and Sher, 2018).

### Computational modeling

GOLD 5.2 (CCDC software; Liebeschuetz *et al.*, 2012) was used to model covalent binding of flucloxacillin to proteins. In order to predict whether flucloxacillin covalent binding could potentially affect protein functions, we have chosen 14-3-3 and liver carboxylesterase 1 representing proteins, in which flucloxacillin bound to the binding sites or nonactive sites, respectively. Crystal structure of 14-3-3 gamma (PDB code 4E2E) and liver carboxylesterase 1 (PDB code 2H7C) (Bencharit *et al.*, 2006) were used to generate models. For covalent docking to lysine residues, the corresponding side chain was removed from the protein and the ligand modified to contain the side chain to allow flexibility. The site of covalent attachment was at the lysine Cα. For HLA-B*57:01 modeling, crystal structure of HLA-B* 57:01 (PDB code 5vuf) (Illing *et al.*, 2018) was used to generate models by removal of the peptide using Pymol (2.0, Schrodinger). The VANALAHKY peptide and its flucloxacillin-modified counterpart were docked within the binding groove, with the binding site defined as 15 Å around the binding point. The binding point was further refined with key amino acid residues within B pocket (Tyr9, Ala24, Met45, Ala46, Glu63, Asn66, Met67, and Tyr99) and F pocket (Asn77, Ile80, Ala81, Ile94. Ile95, Val97, Asp114, and Ser116). A generic algorithm with ChemPLP as the fitness function was used to generate 10 binding modes per ligand. Default settings were retained for the “ligand flexibility,” “fitness and search options,” and “GA settings.”

## Results

### Flucloxacillin cytotoxicity

**Figure 1.**
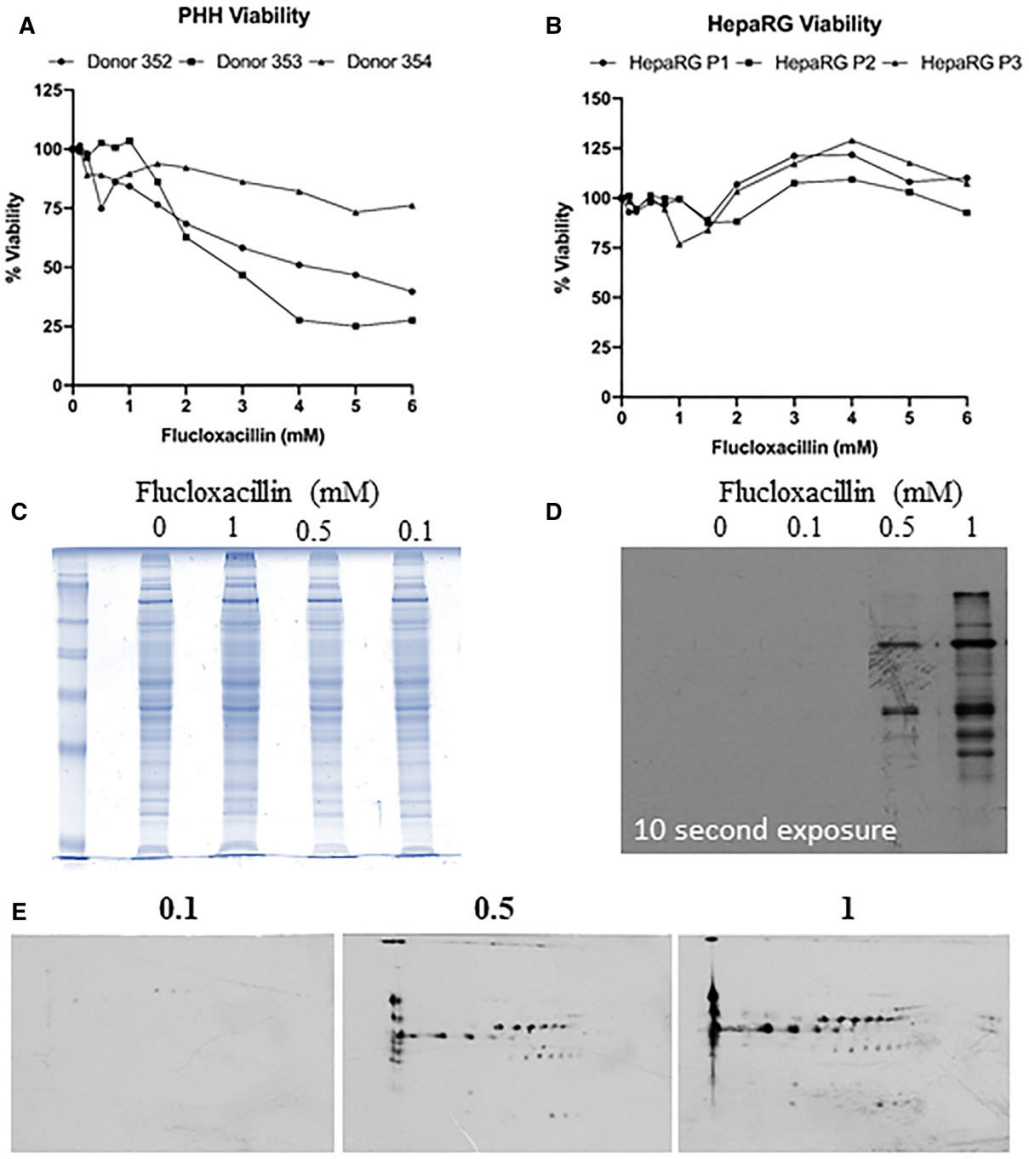
Flucloxacillin modified proteins were detected in human primary hepatocytes. A, The viability of PHHs decreases after flucloxacillin treatment, with approximately 50% reduction in viability between 2 and 3 mM in 2 out of 3 donors. B HepaRGs show no significant reduction in viability upon flucloxacillin treatment. C, PHHs were treated with increasing concentrations of flucloxacillin for 16 h. Coomassie blue staining shows no change in protein abundances upon flucloxacillin treatment. D, A dose-dependent increase in flucloxacillin protein binding was detected using anti-flucloxacillin antibody. E, Multiple flucloxacillin proteins were detected by 2-dimensional Western blot analysis using anti-flucloxacillin antibody.

To determine a nonlethal dose, cellular viability at varying concentrations of flucloxacillin for 24 h was measured using the CellTiter-Glo^®^ Cell Viability Assay. A dose-dependent reduction in flucloxacillin viability was observed in all three donors. No cytotoxicity was observed when PHHs were treated with flucloxacillin at 1.5 mM (2 of 3 PHHs) (Fig. 1A), consistent with our previous observation (Ogese *et al.*, 2017). The concentration of 1.5 mM was therefore chosen for further experimentations on primary hepatocytes based on the following considerations: (1) the long half-lives of flucloxacillin-modified proteins can lead to accumulation of protein adducts in vivo (flucloxacillin-modified albumin in patients is still detectable 30 days post dosage). The levels of protein adducts formed in the in vitro assay at 1 mM are comparable to that measurable in patient plasma (unpublished data); (2) T cells from patients with DILI are optimally activated with mM concentrations of the drugs (Monshi *et al.*, 2013; Wuillemin *et al.*, 2014). This does not mirror HepaRG cells, whereby flucloxacillin can be tolerated up to 6 mM without any reduction in viability (Fig. 1B), possibly due to the higher expression of drug transporters MRP3 and P-gp in HepaRG cells when compared to cryopreserved HPPs (Sison-Young *et al.*, 2017). To further assess whether the covalent binding of flucloxacillin within liver cells is associated with reduction in viability, we treated PHHs with increasing doses of flucloxacillin for 16 h. Posttreatment, cells were lysed to assess intracellular flucloxacillin adduct formation via SDS-PAGE. Interestingly, no significant protein abundance changes were visualized via Coomassie blue staining upon flucloxacillin treatment (Fig. 1C). However, a detailed western blot analysis utilizing a highly specific anti-flucloxacillin antibody demonstrated the dose-dependent increase in flucloxacillin binding (Fig. 1D), mirroring that previously seen in the HepaRG cell line (Waddington *et al.*, 2020a). Additionally, 2D western blot analysis shows the breadth of proteins modified by flucloxacillin over 16 h of treatment with a range of different concentrations of flucloxacillin (Fig. 1E). Although flucloxacillin modification was only detectable when PHHs were treated with 0.5 mM concentration, we have shown that flucloxacillin can form proteins adducts in the plasma from patients received therapeutic doses, indicating covalent binding can occur at nontoxic doses (Jenkins *et al.*, 2009).

### Localization of flucloxacillin modification in primary hepatocytes

**Figure 2.**
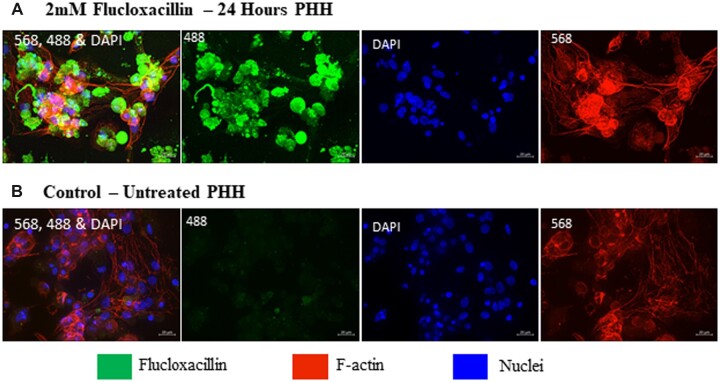
Qualitative assessment of flucloxacillin binding in PHHs. PHHs were treated with 2 mM flucloxacillin for 24 h to visualize localization and binding of flucloxacillin. Although binding of flucloxacillin (488, green) can be visualized within the cells (A) compared to the untreated control (B), the presence or localization at BC is not clearly defined. 40× magnification, scale bar 20 µm.

To further elucidate the localization of flucloxacillin-protein adducts within primary hepatocytes, we employed immunocytochemistry utilizing an anti-flucloxacillin antibody. Although flucloxacillin has been shown to localize within BC regions in the HepaRG cell line (Waddington *et al.*, 2020a), primary hepatocytes do not form BC structures under normal culture conditions. Staining of primary hepatocytes for F-actin and nuclei shows hepatocytes retain their morphology and can maintain a monolayer of cells, however no clearly defined BC structures could be visualized (Fig. 2A). Upon treatment with flucloxacillin for 24 h, flucloxacillin-modified proteins can be detected in primary hepatocytes compared to the control (Fig. 2B). However, the binding could not be localized to any cellular structure.

### Inhibition of flucloxacillin efflux affects covalent binding to hepatic proteins

**Figure 3.**
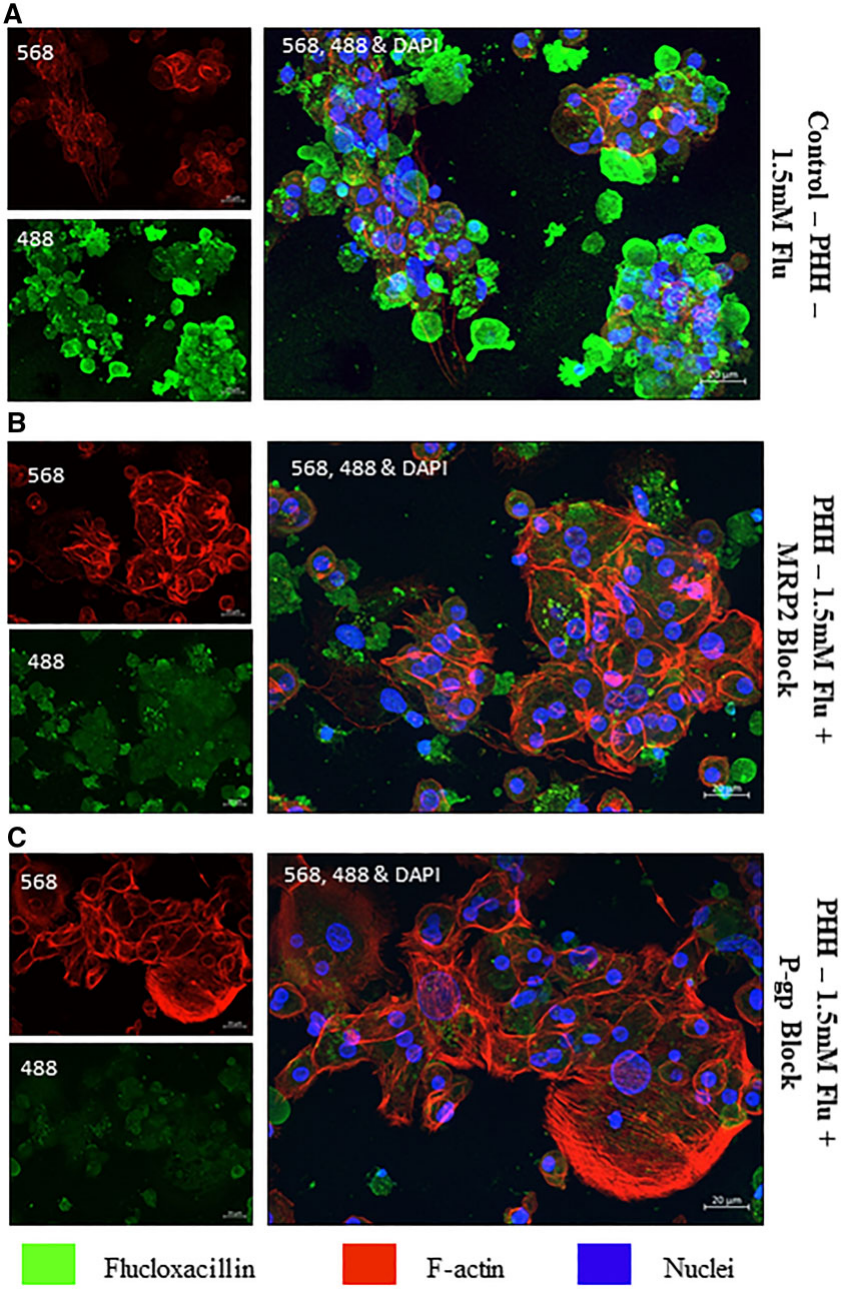
Inhibition of flucloxacillin efflux in PHH. In order to elucidate the role of membrane transporters in transporting flucloxacillin, PHH were treated with 1.5 mM flucloxacillin (488 green) for 24 h with/without inhibitors of transporters. A, Flucloxacillin extensively forms covalent adducts in PHH. Covalent binding of flucloxacillin in PHH was significantly reduced upon the addition of MRP2 inhibitor (MK571, 30 µM) (B) and P-gp inhibitor (valspodar, 12.5 µM) (C). 40× Magnification, scale bar 20 µm.

To investigate the role of membrane transporters in the efflux of flucloxacillin, we cultured PHH with flucloxacillin for 24 h in the presence and absence of MRP2 and P-gp inhibitors MK571 and Valspodar, respectively. Flucloxacillin protein adducts were clearly observed upon drug treatment (Fig. 3A). On the contrary, upon the addition of both MRP2 and P-gp inhibitors, apparent reduction in flucloxacillin binding was observed (Fig. 3B and C). As the images were taken from a small portion of cells at a high magnification (40×), it is important to note that this is a qualitative assessment.

### Flucloxacillin covalently binds to a diverse range of hepatic proteins

To identify potential flucloxacillin-modified proteins in hepatocytes, we used an affinity enrichment-based proteomics approach. Peptides from hepatocytes were first enriched using anti-flucloxacillin antibody from the tryptic digest of hepatocytes, and then analyzed by mass spectrometry. The acquired MS/MS spectra were analyzed by PEAKS software to identify potential flucloxacillin-modified peptides. We used strict manual verification criteria to filter out false positives (Supplementary Fig. 1) to ensure bona fide identification of flucloxacillin-modified peptides. Mass spectrometric analysis of the tryptic digests of flucloxacillin-treated PHH cell lysates revealed over 50 flucloxacillin-modified peptides (Supplementary Table 1), among which 29 flucloxacillin peptides derived from 10 proteins were fully characterized with high confidence (-10lgP > 20, Table 1). Some short flucloxacillin-modified peptides were also detected (Supplementary Table 1), however, the protein source of these modified peptides could not be identified.

**Table 1.** Flucloxacillin modified peptides detected in human primary hepatocytes^a^

Amino acid	**Peptide** ^b^	Protein accession	Protein	-10lgP	ppm	m/z
K50	YK*NVVGAR	P31946/P63104	1433B_HUMAN|14-3-3 protein beta/Alpha/zeta/delta	26.34	3.7	453.8625
K69	EK*GPSVDWGK	Q16851	UGPA_HUMAN|UTP-glucose-1-phosphate uridylyltransferase	29.04	8.4	519.2122
K258	QIAITAGCK*TTTSAVMVHCLR	P23141	EST1_HUMAN| Liver carboxylesterase 1	51.07	−47.9	543.6209
K376	AK*ELIPEATEK			35.06	−3.3	561.2478
K537	K*AVEKPPQTEHIEL			33.26	15.4	691.3257
K134	DYTK*PLEHPPVK	Q8NCE0	SEN2_HUMAN| tRNA-splicing endonuclease subunit Sen2	27.1	−21.6	626.261
R312	PPASQNLSINPMLLLTGR*TWK	P00325	ADH1B_HUMAN|All-trans-retinol dehydrogenase ADH1B	30.53	43	698.3682
K450	ISGASEK*DIVHSGLAY	P00367	DHE3_HUMAN|GDH 1 mitochondrial	52.17	6.3	700.6391
K190	LDELRDEGK*ASSAK	P02768	ALBU_HUMAN|Albumin	55.07	2.6	493.7148
K915	EIGFSDK*QISK	P31327	CPSM_HUMAN|Carbamoyl-phosphate synthase [ammonia] mitochondrial	21.98	−4.2	568.9114
K120	HFGK*EFTPPVQAAYQK	P68871	HBB_HUMAN|Hemoglobin β	57.92	0.9	579.0045
K120	VLAHHFGK*EFTPPVQAAYQK			80.17	2.8	681.0695
K120	AHHFGK*EFTPPVQAAYQK			78.78	−1	628.0288
K120	VCVLAHHFGK*EFTPPVQAAYQK			67.06	5	596.8783
K8	VHLTPEEK*SAV			24.44	−0.2	554.9078
K8	VHLTPEEK*SAVTALWGK			67.08	1.6	773.6954
K82	VLGAFSDGLAHLDNLK*GTFATLSELHCDK			62.29	6.7	717.3376
K95	GTFATLSELHCDK*LHVDPENFR			59.87	−2.1	760.578
K17	SAVTALWGK*VNVDEVGGEALGR			66.5	−1.4	894.411
K95	DK*LHVDPENFR		HBB_HUMAN|Hemoglobin β/HBD_HUMAN|Hemoglobin δ	44.22	3.8	608.2529
K144	VVAGVANALAHK*YH			54.95	2	476.4697
K66	K*VLGAFSDGLAHLDNLK	P68871/P02042		64.91	1.9	751.0227
K90	SALSDLHAHK*LR	P69905	HBA_HUMAN|Hemoglobin α	32.42	−3.9	450.9587
K11	TNVK*AAWGK			49.46	0.3	476.5371
K11	VLSPADKTNVK*AAWGK			37.61	4.1	535.2562
K56	TYFPHFDLSHGSAQVK*GHGK			66.3	2.3	534.0346
K7	VLSPADK*TNVK			28.26	0.8	542.2484
K99	VDPVNFK*LLSH			28.17	−1.1	574.5914
K16	AAWGK*VGAHAGEYGAEALER			91.87	4.3	624.7679

a PHHs from 2 donors were treated with 1.5 mM flucloxacillin for 24 h prior to lysis; cell lysate was digested with trypsin and analyzed by LC-MS/MS.

b Peptide sequences were determined by both manual de novo sequencing and PEAKS X pro 10.6 (Bioinformatics Solution Inc).

* Indicates the flucloxacillin modified residue.

**Figure 4.**
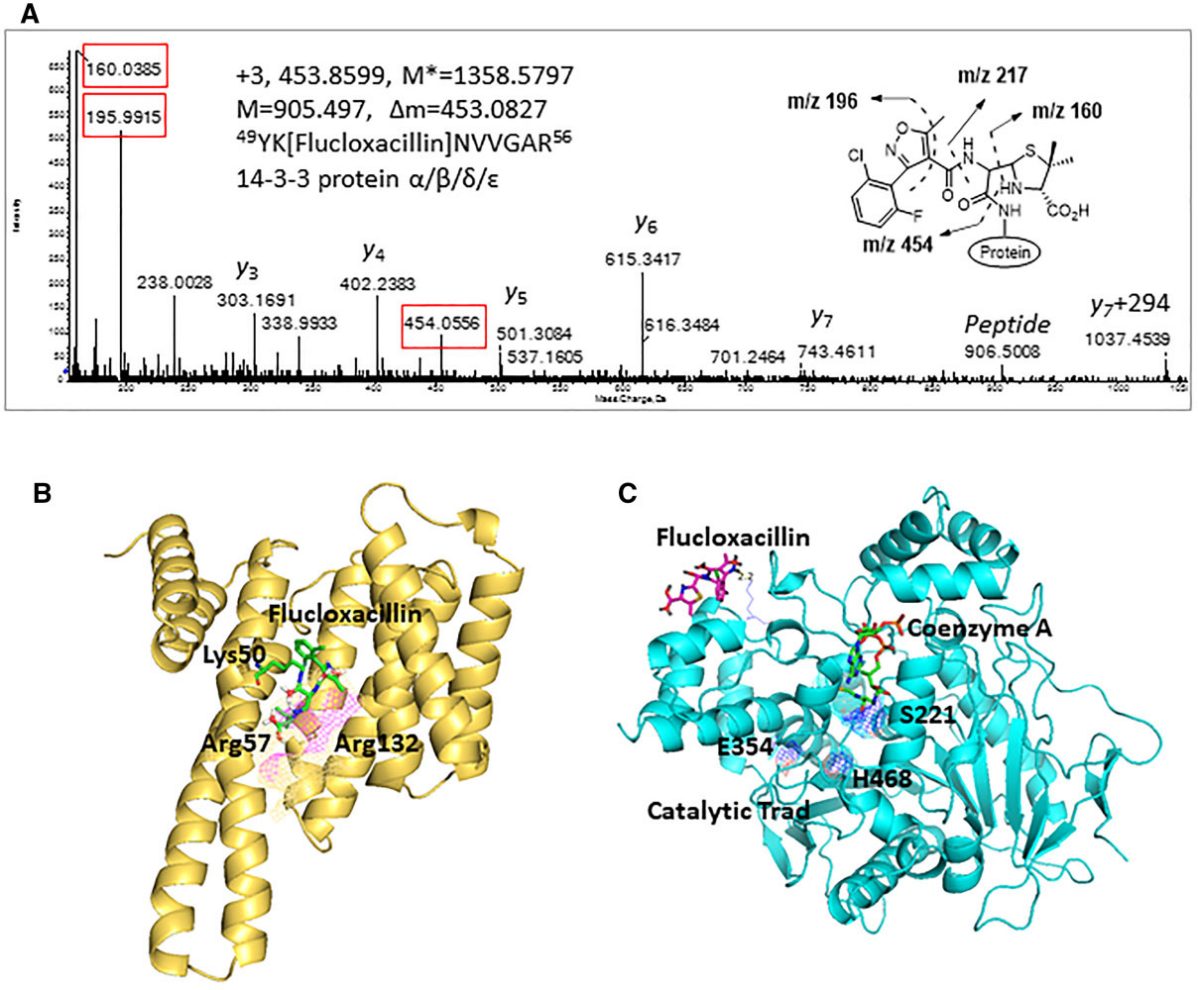
Covalent binding of flucloxacillin to hepatic proteins. A, A representative MS/MS spectrum shows an adduct was formed on 14-3-3 protein (YK[Flucloxacillin]NVVGAR)—M* = observed flucloxacillin-modified peptide mass, M = theoretical unmodified peptide mass. Characteristic fragment ions from flucloxacillin are highlighted (red boxes). B, Molecular modeling predicts that covalent binding of flucloxacillin to Lys50 on 14-3-3 proteins (PDB code 4E2E) would clash with Arg57 and Arg132 (purple mesh), the key amino acids involved in phosphorylation of binding partner proteins. C, Flucloxacillin (purple) covalently binds to multiple lysine residues on liver carboxylesterase 1, which are distant from the catalytic domain (blue mesh, PDB code 2H7C) and the ligand (coenzyme A, green) binding pocket. Images are illustrated by PyMOL (The PyMOL Molecular Graphics System, Version 1.3 Schrödinger, LLC.).

A diverse range of proteins with different functions was found to be targeted by flucloxacillin, including adaptor proteins (14-3-3), proteins with catalytic activities (liver carboxylesterase 1, tRNA-splicing endonuclease subunit Sen2, All-trans-retinol dehydrogenase ADH1B, Glutamate dehydrogenase [GDH] 1 mitochondrial, Carbamoyl-phosphate synthase [ammonia] mitochondrial), and transporters (hemoglobin, albumin, and UTP-glucose-1-phosphate uridylyltransferase). One important flucloxacillin-modified protein is 14-3-3, a family of phosphoprotein-binding proteins which regulate major cellular functions including cell cycle progression and apoptosis. Flucloxacillin-modified 14-3-3 peptide ^49^YK[flucloxacillin] NVVGAR^56^ was detected by mass spectrometry analysis. Figure 4A shows a representative MS/MS spectrum for a triply charged ion of m/z 453.8599, which corresponds to the tryptic peptide ^49^YKNVVGAR^56^ with a mass addition of 453.0827 Da, indicating the presence of flucloxacillin. The presence of protonated flucloxacillin (m/z 454.0556) and characteristic fragment ions (m/z 160.0385 and m/z 195.9915) derived from flucloxacillin fragmentation during collision-induced dissociation provided further evidence of modification. The modification site (Lys50) was confirmed by the presence of flucloxacillin-modified y7 ion (m/z 1037.4539), which has a mass addition of 294 Da, corresponding to the mass of the adduct after cleavage of the thiazolidine ring fragment (Fig. 4A).

Covalent binding of those proteins with catalytic functions can potentially regulate their downstream functions. Computational modeling studies predicted that covalent binding of flucloxacillin to Lys50, one of 3 positively charged residues (Lys50, Arg57, and Arg132) within the 14-3-3 protein phospho-binding pocket, would lead a clash between flucloxacillin and Arg57, which could block its binding to signaling proteins (Fig. 4B). On the contrary, all 3 binding sites on liver carboxylesterase 1 (Lys258, Lys376, and Lys537) are distant from the catalytic trad (Glu354, Ser221, and His468) and the ligand binding pocket (Fig. 4C). Covalent binding to these sites may not have a direct impact on the protein functions.

### Flucloxacillin predominately targets hemoglobin in hepatocytes

**Figure 5.**
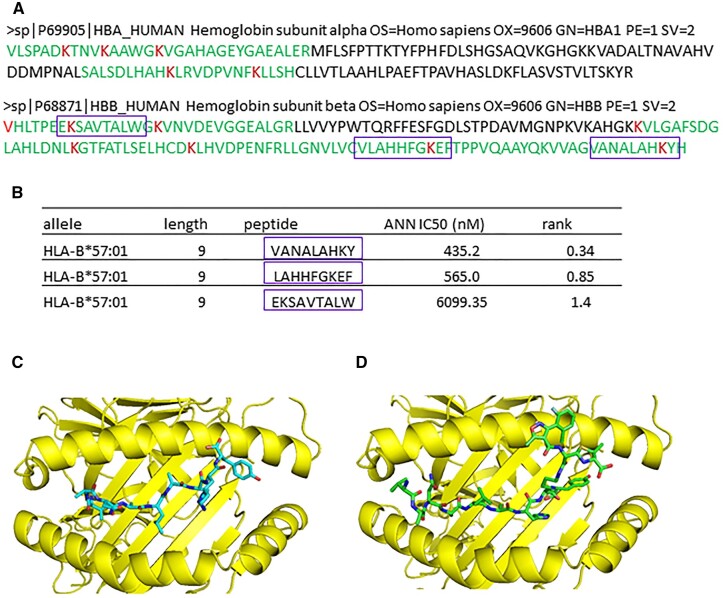
Flucloxacillin predominately targets hemoglobin in human hepatocytes. A, Six out of 11 total lysine residues on hemoglobin subunit α and 7 out of 11 total lysine residues on hemoglobin subunit β were modified by flucloxacillin (modified lysine residues are highlighted in red and peptides sequence are in green). B, Some hemoglobin peptides containing modified lysine residues are also good binders to HLA-B*57:01, a risk allele associated with flucloxacillin-induced liver injury (purple boxes). C, In silicon modeling shows peptide VANALAHKY derived from hemoglobin binds to the binding groove of HLA-B*57:01(PDB code 5vuf, Illing *et al.*, 2018), with P9 (Y) occupying the F pocket. D, The predicted conformation of flucloxacillin-haptenated VANALAHK[flucloxacillin]Y is different from the native peptide, with flucloxacillin molecule reaching out of the binding groove, available for T cell recognition. Images are illustrated by PyMOL (The PyMOL Molecular Graphics System, Version 1.3 Schrödinger, LLC.).

One major protein within PHH targeted by flucloxacillin was hemoglobin. It appears both α and β chains of hemoglobin can be targeted by flucloxacillin, with 6 out of 11 total lysine residues on the α chain and 7 out 11 total lysine residues on the β chain being modified. It is important to note that some of these flucloxacillin-modified lysine residues are only detectable after immunoaffinity enrichment, indicating they may be present at very low levels compared to unmodified hemoglobin (Fig. 5A). In silico analysis predicted that some flucloxacillin-modified hemoglobin peptides were good binders to HLA-B*57:01 (http://tools.iedb.org/mhci/; last accessed February 15, 2023; Fig. 5B). HLA-B*57:01 is the antigen-presenting molecule associated with flucloxacillin-induced liver injury (Daly *et al.*, 2009). The predicted binding affinity of peptide VANALAHKY was high (IC50 = 435.2 nM), indicating it is a strong binder to HLA-B*57:01 (IC50 values of <500 nM for binding to HLA molecules were considered as good binders) (Zhao and Sher, 2018). Computational modeling of Hemoglobin peptide VANALAHKY with HLA-B*57:01 (PDB code 5vuf) demonstrated that the peptide could be accommodated into the binding groove, with P9 (Y) occupying the F pocket (Fig. 5C). On the other hand, the predicted conformation of flucloxacillin-modified VANALAHK[flucloxacillin]Y is different from the native peptide, with the flucloxacillin molecule reaching out of the binding groove, potentially making it available for T-cell recognition (Fig. 5D).

## Discussion

Flucloxacillin-DILI is multifactorial, with both immune-mediated and nonimmune-mediated factors partaking in disease progression (Burban *et al.*, 2017; Daly *et al.*, 2009; Monshi *et al.*, 2013; Wuillemin *et al.*, 2014). Individual variation in normal physiological processes or disruption by flucloxacillin may be the trigger for cell stress and immune activation. Given the cholestatic nature of flucloxacillin, we aimed to examine the effect of flucloxacillin on membrane transporters using an anti-flucloxacillin antibody and identify cellular targets for flucloxacillin binding using mass spectrometry analysis.

We first assessed the cytotoxic capabilities of flucloxacillin to ensure doses utilized in this study did not result in the deformation of cells used. We found that flucloxacillin was not toxic to PHHs and HepaRGs over a 24-h period at concentrations (1.5 mM) utilized previously in both T-cell assays and imaging experimentation (Monshi *et al.*, 2013; Ogese *et al.*, 2017; Waddington *et al.*, 2020a,b). It is important to note that the drug concentrations used in in vitro assays are apparently higher than the reported Cmax in humans (Landersdorfer *et al.*, 2007), indicating flucloxacillin is not intrinsically toxic to hepatocytes in vivo (Burban *et al.*, 2017). Thereby, this lack of intrinsic toxicity suggests flucloxacillin may initiate cell stress signaling via an alternative pathway. The hepatobiliary transport system, especially bile acid transporters, play a crucial role in the promotion of cholestatic diseases (Sharanek *et al.*, 2016; Stieger, 2010; Welch *et al.*, 2015). These transporters located in the canalicular or apical membrane of hepatocytes maintain the transport systems required for bile salt movement. Under normal physiological conditions, toxic levels of bile salts are kept low through canicular bile flow, mediated by the important transport mechanisms. Alteration of such mechanisms, could reduce bile flow, resulting in cholestasis and subsequent cytotoxic effects (Li *et al.*, 2017; Stieger, 2010). We therefore employed immunocytochemistry to examine the effect of flucloxacillin on transporters using HepaRG cells and PHHs. Previously we found flucloxacillin conjugating in the tight actin bundles after treating HepaRGs with increasing concentrations of flucloxacillin, suggesting localization at BC, which was further confirmed by staining for MRP2 and P-gp within BC regions (Waddington *et al.*, 2020a) Prolonged flucloxacillin treatment of HepaRGs has resulted in significant BC dilation, consistent with published findings (Burban *et al.*, 2017). The effect of flucloxacillin on the activity of the transporter was further examined using PHHs. Although primary hepatocytes do not form BC structures in 2D culture in vitro, membrane transporter proteins such as MRP2 and P-gp are expressed at functionally relevant levels during initial culture. It appears that covalent binding of flucloxacillin was reduced upon inhibition of MRP2 and P-gp, indicating these transporters were involved in cellular flucloxacillin disposition As the exact location (intracellular/BC) of covalent binding cannot be determined in 2D culture, future work using primary hepatocytes in 3D spheroid culture would hopefully provide clearer results.

Flucloxacillin has been shown to bind to human albumin in patient plasma (Jenkins *et al.*, 2009), cellular proteins in B-cells, and HLA-B*57:01 on the surface of antigen-presenting cells (Waddington *et al.*, 2020b). Each of these adducts could potentially serve as direct or indirect antigens for T-cell activation. Indeed, circulating flucloxacillin-specific T-cells, activated via a hapten pathway, have been detected in patients with DILI (Monshi *et al.*, 2013). However, the local antigens formed by flucloxacillin in the liver remain to be defined. In this study, we found flucloxacillin extensively bound to hepatic proteins, particularly hemoglobin α and β, with 14 out of 22 lysine residues being modified. In total 10 flucloxacillin-modified hepatocellular proteins were identified. The majority of proteins modified with flucloxacillin are involved in molecular functions including catalytic activities (liver carboxylesterase 1, tRNA-splicing endonuclease subunit Sen2, All-trans-retinol dehydrogenase ADH1B, GDH 1 mitochondrial, Carbamoyl-phosphate synthase [ammonia] mitochondrial), binding and transport (hemoglobin, albumin, and UTP-glucose-1-phosphate uridylyltransferase), and cellular signaling (14-3-3). Of particular interest, flucloxacillin adducted Lys50 on 14-3-3 proteins, which has previously been characterized in human B-cells, was also detected in hepatocytes, indicating that 14-3-3 proteins could be a major cellular target for flucloxacillin (Waddington *et al.*, 2020b). The 14-3-3 proteins are phospho-binding proteins that regulate major cellular functions including cell proliferation, growth, apoptosis, autophagy, and cell motility (Pennington *et al.*, 2018). Of note, Lys50 is one of three positively charged residues (Lys50, Arg57, and Arg132) important for binding to phosphorylated proteins. Modeling of flucloxacillin covalent binding to Lys50 on 14-3-3 predicted a clash between flucloxacillin and Arg57, which could block its binding to partner proteins. However, since 14-3-3 proteins are involved in a wide range of regulatory processes by binding to more than 200 partner proteins (Sun *et al.*, 2009), the specific regulatory process which may be altered by flucloxacillin modification remains unknown. This warrants future studies to identify downstream 14-3-3 regulatory processes that may be involved in flucloxacillin-induced liver injury.

Flucloxacillin was also found to target a variety of proteins with catalytic activity, particularly mitochondrial proteins involved in metabolism of both endogenous compounds and xenobiotics. Covalent binding to these proteins could potentially affect their functional activity, leading to cellular stress. For example, liver carboxylesterase 1 (CES1) plays an important role in the metabolism of a wide range of endogenous esters and ester-containing drugs (Bencharit *et al.*, 2006; Wang *et al.*, 2018). Flucloxacillin was found to bind to three sites (Lys258, Lys376, and Lys537) on CES1, which are distant from the catalytic domain (catalytic trad: Glu354, Ser221, and His468) and the substrate binding pocket (Fig. 4C). Despite this, flucloxacillin modification may change the conformation of CES1, allowing the enzyme to perform distinct catalytic actions (Bencharit *et al.*, 2006). Interestingly, flucloxacillin was found to bind to two mitochondrial proteins that have been identified as promising biomarkers of liver injury. Carbamoyl-phosphate synthase (CPS), the most abundant mitochondrial matrix protein in hepatocytes has an important role in removing excessive cellular ammonia and has been identified as a biomarker for apoptotic and necrotic forms of hepatocyte death and injury (Weerasinghe *et al.*, 2014). Flucloxacillin was found to bind to Lys915 on CPS that is located within the integrating domain. Although the modification site is distant from the substrate binding and catalytic machinery of CPS, modification could trigger integrating domain misfolding, leading to distorted interaction of the integrating domain with other protein domains (Diez-Fernandez *et al.*, 2014). More importantly, since CPS has been shown previously to be released into the blood and taken up by monocytes upon liver injury (Park *et al.*, 2019), flucloxacillin-modified CPS could interact with liver-resident and circulating immune cells, leading to activation of flucloxacillin-specific T-cells. Another mitochondrial protein targeted by flucloxacillin is GDH, which is responsible for the reversible interconversion of glutamate to a-ketoglutarate and ammonia. However, the function of flucloxacillin-modified GDH is yet to be investigated.

One particular protein targeted by flucloxacillin was hemoglobin. For this reason, the HLA-B*57:01 binding of flucloxacillin-modified peptides derived from hemoglobin was modeled. Several peptides predicted as having high binding affinity to HLA-B*57:01 contain lysine residues found to be modified by flucloxacillin, which could ultimately result in activation of drug-specific T-cells. Indeed, computational modeling of flucloxacillin modified hemoglobin peptide VANALAHK[Flucloxacillin]Y with HLA-B*57:01 demonstrated that this peptide could be accommodated into the binding groove, with the flucloxacillin molecule reaching out of the binding groove available for T-cell recognition. Further experimental work is needed to determine whether these potential HLA-B*57:01 ligands derived from flucloxacillin-modified hemoglobin can be presented by hepatocytes. Assessing the immunogenicity of these ligands in patients with a history of flucloxacillin DILI will be pivotal to our understanding of immunopathology of flucloxacillin-induced liver injury. However, the majority of peptides containing flucloxacillin modification were not predicted as good binders to HLA-B*57:01, indicating covalent binding may alter other functions. Further investigation of whether flucloxacillin modification could affect the functions of hemoglobin will help define the roles of haptenated hemoglobin in flucloxacillin-induced cholestatic liver injury.

Together these data indicate that flucloxacillin extensively modifies PHH protein, with the localization and accumulation of flucloxacillin being amplified as a result of differences in MRP2 and P-gp expression. Consequently, the destruction of BC structures and damage to other local hepatic cells may be an important early “danger” trigger for the disruption of normal hepatic tolerance mechanisms. Although the exact localization of flucloxacillin covalent binding cannot be defined using PHHs in this study, we have shown MRP2 and P-gp transporters could be potentially involved in flucloxacillin cellular disposition. We speculated that the accumulation of flucloxacillin within liver tissue as a result of membrane transporter distress may result in the increased formation of neo-antigens. Incorporation of these neo-antigens into T-cell assays would help decipher the propensity of such peptides to activate T-cells. Likewise, the usage of an autologous liver model incorporating these danger signals, alongside immune cell subsets and liver tissue may help provide a panacea to understanding flucloxacillin-DILI.

## Supplementary Material

kfad015_Supplementary_Data
