# Supplementary material for: Identification of flucloxacillin-modified hepatocellular proteins: implications in flucloxacillin-induced liver injury
Source: Toxicol Sci. 2023 Feb 14;192(1):106–16. doi: 10.1093/toxsci/kfad015 (PMC10371196; doi:10.1093/toxsci/kfad015)
Supplement: kfad015_Supplementary_Data [file kfad015_supplementary_data.pdf]

Identification of flucloxacillin-modified hepatocellular proteins: Implication in flucloxacillin-induced liver injury

Serat-E Ali†, James C. Waddington†, Adam Lister†, Rowena Sison-Young†, Robert P Jones‡, Adeeb H Rehman‡, Chris E.P. Goldring†, Dean J. Naisbitt†, Xiaoli Meng†*

†Dept. Molecular & Clinical Pharmacology, University of Liverpool, Sherrington Buildings, Ashton Street, Liverpool, L69 3GE, UK.
‡Department of Hepatobiliary Surgery, Aintree University Hospital, Liverpool University Hospitals, NHS Foundation Trust, Liverpool UK.
Corresponding Author: Dr Xiaoli Meng
Department of Pharmacology, University of Liverpool, Sherrington Building, Ashton Street, Liverpool L69 3GE, England
Telephone: 0044 151 7956066; e-mail: xlmeng@liverpool.ac.uk

Figure S1

A

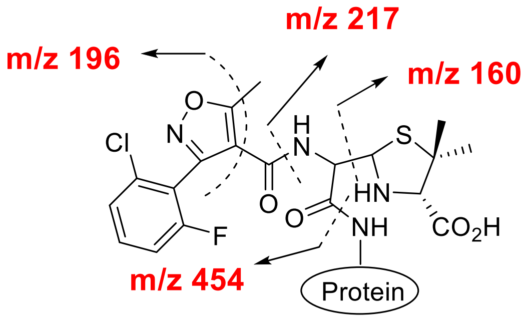

B

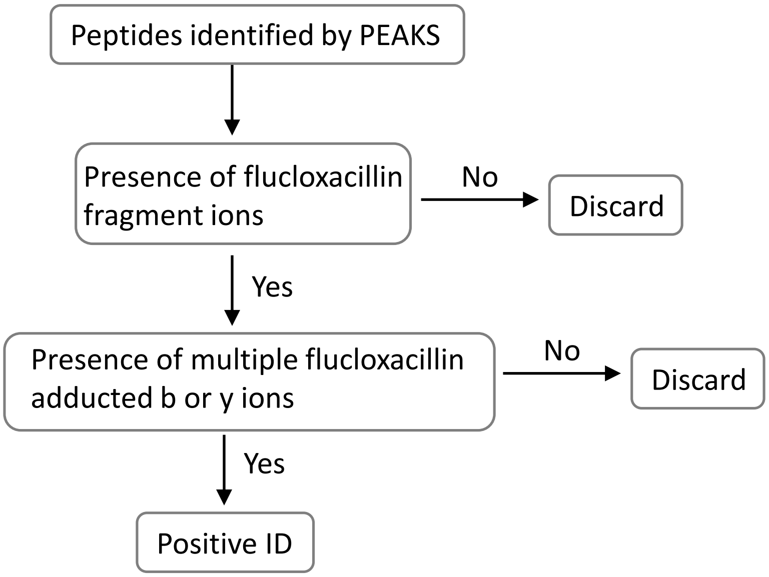

Figure S1. Manual verification to identify flucloxacillin modified peptides. (A) characteristic fragment ions derived from flucloxacillin. (B) Verification criteria to filter out false positive identification of flucloxacillin modified peptides.

Table S1. Flucloxacillin-modified peptides detected in primary human hepatocytes

m/z	Peptide	Protein Accession	-10lgP	Characteristic fragment ions
698.3682	PPASQNLSINPMLLLTGR*TWK	P00325	30.53	160, 196, 454
700.6391	ISGASEK*DIVHSGLAY	P00367	52.17	160, 196, 454
493.7148	LDELRDEGK*ASSAK	P02768	55.07	160, 196, 454
543.6209	QIAITAGCK*TTTSAVMVHCLR	P23141	51.07	160, 196, 454
561.2478	AK*ELIPEATEK	P23141	35.06	160, 196, 454
691.3257	K*AVEKPPQTEHIEL	P23141	33.26	160, 196, 454
568.9114	EIGFSDK*QISK	P31327	21.98	160, 196, 454
453.8625	YK*NVVGAR	P31946/P63104	26.34	160, 196, 454
554.9078	VHLTPEEK*SAV	P68871	24.44	160, 196, 454
579.0045	HFGK*EFTPPVQAAYQK	P68871	57.92	160, 196, 454
596.8783	VCVLAHHFGK*EFTPPVQAAYQK	P68871	67.06	160, 196, 454
628.0288	AHHFGK*EFTPPVQAAYQK	P68871	78.78	160, 196, 454
681.0695	VLAHHFGK*EFTPPVQAAYQK	P68871	80.17	160, 196, 454
717.3376	VLGAFSDGLAHLDNLK*GTFATLSELHCDK	P68871	62.29	160, 196, 454
760.578	GTFATLSELHCDK*LHVDPENFR	P68871	59.87	160, 196, 454
773.6954	VHLTPEEK*SAVTALWGK	P68871	67.08	160, 196, 454
894.411	SAVTALWGK*VNVDEVGGEALGR	P68871	66.5	160, 196, 454
476.4697	VVAGVANALAHK*YH	P68871/P02042	54.95	160, 196, 454
608.2529	DK*LHVDPENFR	P68871/P02042	44.22	160, 196, 454
751.0227	K*VLGAFSDGLAHLDNLK	P68871/P02042	64.91	160, 196, 454
450.9587	SALSDLHAHK*LR	P69905	32.42	160, 196, 454
476.5371	TNVK*AAWGK	P69905	49.46	160, 196, 454
534.0346	TYFPHFDLSHGSAQVK*GHGK	P69905	66.3	160, 196, 454
535.2562	VLSPADKTNVK*AAWGK	P69905	37.61	160, 196, 454
542.2484	VLSPADK*TNVK	P69905	28.26	160, 196, 454
574.5914	VDPVNFK*LLSH	P69905	28.17	160, 196, 454
624.7679	AAWGK*VGAHAGEYGAEALER	P69905	91.87	160, 196, 454
481.5441	DLHAAK*LR	P69905	NA	160, 196, 454
519.2122	EK*GPSVDWGK	Q16851	29.04	160, 196, 454
626.261	DYTK*PLEHPPVK	Q8NCE0	27.1	160, 196, 454
525.7	K*LLSH	Q96JJ3	NA	160, 196, 454
429.1625	EK*K	NA	NA	160, 196, 454
435.1799	K*LR	NA	NA	160, 196, 454
443.1617	EK*R	NA	NA	160, 196, 454
414.1673	VK*K	NA	NA	160, 196, 454
421.1744	K*LK	NA	NA	160, 196
421.6566	K*NK	NA	NA	160, 196
465.2247	NA	NA	NA	160, 196, 454
469.203	NA	NA	NA	160, 196, 454
459.2277	NA	NA	NA	160, 196, 454
477.2	NA	NA	NA	160, 196, 454
490.5	NA	NA	NA	160, 196, 454
512.6	NA	NA	NA	160, 196, 454
545.1	NA	NA	NA	160, 196, 454
564	NA	NA	NA	160, 196, 454
573.8	NA	NA	NA	160, 196, 454
580.5	NA	NA	NA	160, 196, 454
590	NA	NA	NA	160, 196, 454
591.7	NA	NA	NA	160, 196, 454
593	NA	NA	NA	160, 196, 454
600.3	NA	NA	NA	160, 196, 454
617	NA	NA	NA	160, 196, 454
621	NA	NA	NA	160, 196, 454

Table S2. Predicted binding of flucloxacillin modified peptides to HLA-B*57:01

Amino acid	Flucloxicillin modified Peptides[1]	9-mer peptide	NetMHCpan score	Percentile Rank	ANN IC50(nM)	Binding affinity	Protein Accession
Lys503	ISGASEK*DIVHSGLAY	ISGASEKDI	0.004068	9.8	3936.17	low	P00367
Lys190	LDELRDEGK*ASSAK	RDEGKASSA	6.40E-05	66	34669.86	non-binder	P02768
Lys915	EIGFSDK*QISK	GFSDKQISK	0.000372	29	26774.24	non-binder	P31327
Lys9	VHLTPEEK*SAV	LTPEEKSAV	0.001982	14	24989.98	non-binder	P68871
Lys83	VLGAFSDGLAHLDNLK*GTFATLSELHCDK	HLDNLKGTF	0.024	4.2	17898.12	non-binder	P68871
Lys121	LLGNVLVCVLAHHFGK*EFTPPVQAAYQK	LAHHFGKEF	0.3319	0.85	565.0	low	P68871
Lys96	GTFATLSELHCDK*LHVDPENFR	KLHVDPENF	0.09	1.9	13318.93	non-binder	P68871
Lys18	SAVTALWGK*VNVDEVGGEALGR	TALWGKVNV	0.00698	7.6	23395.4	non-binder	P68871
Lys145	VVAGVANALAHK*YH	VANALAHKY	0.65943	0.34	435.2	intermediate	P68871/P02042
Lys67	K*VLGAFSDGLAHLDNLK	KVLGAFSDG	0.000535	25	16943.48	non-binder	P68871/P02042
Lys91	SALSDLHAHK*LR	LSDLHAHKL	0.1504	1.6	9815.15	non-binder	P69905
Lys12	TNVK*AAWGK	TNVKAAWGK	0.000713	22	29091.6	non-binder	P69905
Lys57	TYFPHFDLSHGSAQVK*GHGK	LSHGSAQVK	0.008756	6.8	17170.10	non-binder	P69905
Lys12	VLSPADKTNVK*AAWGK	DKTNVKAAW	0.024557	4.1	20681.68	non-binder	P69905
Lys8	VLSPADK*TNVK	LSPADKTNV	0.002515	13	20783.07	non-binder	P69905
Lys100	VDPVNFK*LLSH	PVNFKLLSH	0.000781	22	28166.7	non-binder	P69905
Lys17	AAWGK*VGAHAGEYGAEALER	KVGAHAGEY	0.03334	3.6	11673.42	non-binder	P69905

50 nM are considered high affinity, <500 nM intermediate affinity and <5000 nM low affinity
The MHCI binding was predicted using the IEDB analysis resource NetMHCpan (ver. 4.1) tool [1].

Birkir Reynisson, Bruno Alvarez, Sinu Paul, Bjoern Peters, Morten Nielsen. 2020. NetMHCpan-4.1 and NetMHCIIpan-4.0: improved predictions of MHC antigen presentation by concurrent motif deconvolution and integration of MS MHC eluted ligand data. Nucleic Acids Res. 48(W1):W449-W454. doi: 10.1093/nar/gkaa379.
